# Quadriceps performance under activation of foot dorsal extension in healthy volunteers: an interventional cohort study

**DOI:** 10.1186/s12891-015-0774-0

**Published:** 2015-11-06

**Authors:** Felix Angst, Martina Kaufmann, Thomas Benz, Stefan Nehrer, André Aeschlimann, Susanne Lehmann

**Affiliations:** Research Department, Rehabilitation Clinic “RehaClinic”, Quellenstrasse 34, 5330 Bad Zurzach, Switzerland; Donau-University, Krems, Austria

**Keywords:** Dorsal foot extension, Quadriceps, Muscle activation, Performance, Electromyography, Training, Rehabilitation, Standardized response mean

## Abstract

**Background:**

The m. quadriceps femoris is the strongest muscle in the human body and plays an important role in sports, activities of daily living and independence. Two older studies showed increased electromyographic (EMG) activity of the quadriceps when the dorsal extensors of the foot were pre-activated. The aim was to physiologically replicate this finding by EMG and to verify it functionally by single leg hop.

**Methods:**

EMG activity (root mean square, RMS) was tested on the leg press at the isometric load of the individual 12-repetition-maximum (12RM) weight (on average 79.7 kg) at 45° and 90° knee flexion. Single leg hop distance was measured between the tests. Intra-individual changes between with and without dorsal foot extension were quantified and compared by standardized response means (SRM).

**Results:**

Thirty-five healthy subjects between 21 and 57 years were included. The m. vastus medialis was activated on average to an RMS of 32.4 μV without and 53.7 μV with dorsal foot extension (SRM = 1.39, p < 0.001) at 45° knee flexion and an RMS of 124.9 μV versus 152.8 μV (SRM = 1.08, *p* < 0.001) at 90°. The corresponding data for the rectus femoris were 9.4 μV versus 18.9 μV (SRM = 0.71, *p* < 0.001) at 45° and 77.8 μV versus 135.3 μV (SRM = 0.89, *p* < 0.001) at 90°. Mean single leg hop distance was 169.8 cm without versus 178.9 cm with dorsal foot extension (SRM = 1.09, *p* < 0.001).

**Conclusions:**

Pre-activation of dorsal foot extensors significantly increased EMG activity in the m. quadriceps femoris and single leg hop distance. It can therefore be used to improve functional quadriceps muscle performance and knee joint stability in training and rehabilitation.

## Background

The biggest and most powerful muscle of the human body, the m. quadriceps femoris, weighs around 2 kg and has a cross-sectional surface area of more than 180 cm^2^ [1]. During walking and jumping, it extends the knee joint (concentric contraction) and decelerates the impact of body weight during landing (excentric contraction) [2]. Besides these important functions of mobility, it stabilizes the knee joint, which is important for the ability and confidence to stand safely [3, 4]. Quadriceps performance in terms of functional knee stability is crucial in controlling the body's momentum during landing after a jump or step and plays a major role in sport performance as well as in the control of symptoms in knee osteoarthritis [2, 5]. Quadriceps training improves muscle performance during walking, jumping, and lifting weights [4, 6].

Immobilization and increasing age rapidly reduce muscle force, especially of the quadriceps [7]. Knee osteoarthritis is a further important cause of quadriceps weakness [8]. It leads to relatively higher EMG activity of the ischiocrural muscles when compared to that of the quadriceps [8]. The quadriceps femoris atrophies twice as fast as the ischiocrural musculature [9]. The latter functional group consists of the m. biceps femoris, m. semimembranosus, and m. semitendinosus. After 10 days of immobilization, cross-sectional surface area of the quadriceps decreases by 12 % and strength by 40 % [9]. Osteoarthritis and knee arthroplasty as well as low back pain reduce quadriceps performance and lead to relative electromyographic (EMG) overactivity of the antagonistic ischiocrural muscle group (muscular dysbalance) [8, 10].

In sports and rehabilitation, squats, lunges, leg extension and leg press are the most important modalities in quadriceps training [4]. To optimize training, activity of the quadriceps is quantified by EMG and the recorded values taken to indicate the extent of stress/load on the muscle as a surrogate [11, 12]. The relationship between EMG activity and torque of the muscle was determined to be linear and the relationship between EMG activity and isometric strength was quadratic [11].

Two older studies showed that dorsal foot extension increased EMG activity of the quadriceps femoris [13, 14]. On the leg press, EMG activity of the rectus femoris was highest if the foot was extended dorsally, followed by neutral position of the foot and plantar flexion [14]. Voluntary isometric quadriceps contraction activity was maximal in dorsal extension of the foot, somewhat but not significantly smaller than in plantar flexion, and significantly smallest (compared to both positions) when the foot was in the neutral position/at rest [13]. However, both studies had small sample sizes (*n* = 10 and *n* = 20) and did not perform any a priori power calculation or sample size determination. Furthermore, EMG activity was not related to functional performance. Specifically, the functional effect of dorsal foot extension on any functional performance was not examined in either study.

The first aim of this study was to quantify change of quadriceps EMG activation with and without foot dorsal extension, specifically for the rectus femoris and the vastus medialis by simultaneous application of isometric load on the leg press. The second aim was to examine and to quantify change of single leg hop distance with and without foot dorsal extension to link the EMG surrogate to functional quadriceps performance.

## Methods

### Study design and subjects

In this monocentric cross-sectional pilot study, healthy volunteers were recruited at the rehabilitation clinic (“RehaClinic”), Bad Zurzach, Switzerland (orthopedic and neurological rehabilitation). First, all employees of the clinic were informed and asked to participate by e-mail. Recruiting was stopped when the predetermined minimum sample size was reached (see Analysis). Inclusion criteria were healthy subjects between 18 and 60 years and agreement to participate by informed consent. Excluded were persons having musculoskeletal, neurological, or joint disease. No financial or other recompense was given. The project was approved by the local ethics committee, the “Kantonale Ethikkommission (KEK) des Kanton Aargau (AG)” (cantonal ethic committee of the canton Aargau, Switzerland), KEK AG 2010/063.

### Test procedures

**Fig. 1:**
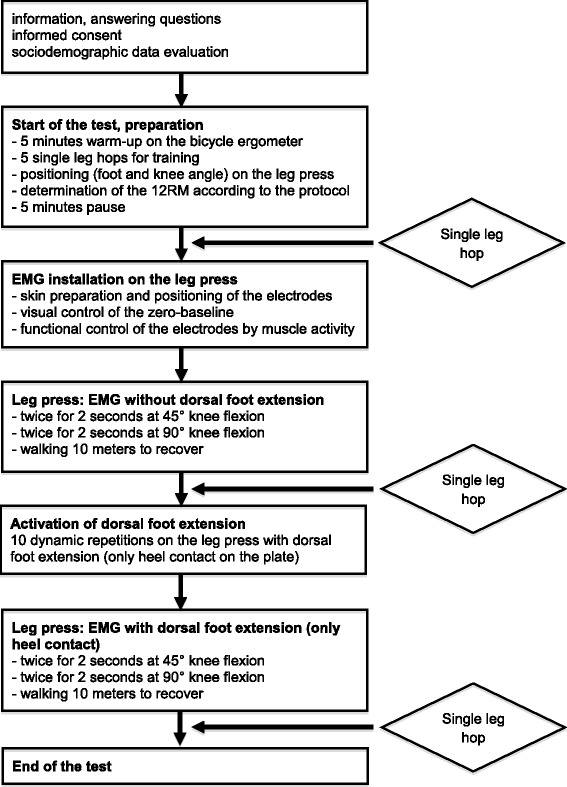
Test procedure

The test procedure was performed as described in the flowchart of Fig. 1. EMG testing on the leg press was performed at angles of 45°and 90° of knee flexion. Between 0° and 45°, stress on the femoropatellar joint is minimized and safety for the subject is high [3, 4]. At 45° knee flexion, rectus femoris and biceps femoralis do not only act as antagonists but also as synergists [15]. Leg press at 90° knee flexion has been performed in several previous studies that provide comparative data [7, 14]. For pre-activation of dorsal foot extension, the subjects had to walk 10 steps on the heel. On the leg press, dorsal foot extension was performed by each individual on the command of the examiner without any supports or assistance. It was ensured that only the heel and not the forefoot contacted the plate on the leg-press. To control that dorsal foot extension was really performed and to quantify it during testing (with compared to without foot dorsal extension), the EMG was derived from the m. tibialis anterior. Since detailed EMG analysis of the single leg hop was not a main focus of the study (the main focus being on the quadriceps, the most important knee extensor), and the m. gastrocnemius (antagonist) is relaxed under dorsal foot extension, this muscle was not examined by EMG.

The 12-repetition maximum (12RM) weight was identified per individual and defined as the load on the leg press so that the current findings could be compared with previous EMG recordings and training settings [3, 16]. It is used in most of the comparable studies. The 12RM is the preinstalled weight that the subject is able to press 12 times at maximum. The second reason to prefer the 12RM was to be closer to realistic muscle training that generally recommends 10–20 repetitions rather than 1 repetition (1RM), for example [3]. The 12RM corresponded to 67 % of the 1RM and was determined by standardized protocol [17]. After initial estimation, it was tested in 2–3 passes where the weight was successively changed by 10-20 % with recovery pauses of 2 min each between the tests.

After holding the correct knee flexion angle and the correct foot position on the plate, the proband had to press the isometric 12RM on the leg press. During this activity, EMG was recorded twice in 2 s. The average of the two EMG measurements was taken for further analysis.

### Outcome measurement

The EMG recorder was rented from Prophysics, Switzerland (registered trade mark). It had the capacity to measure all four muscles simultaneously, the m. tibialis anterior, m. biceps femoris, m. vastus medialis, and m. rectus femoris. The sum of all activated muscle fibers, i.e. motoneural units by depolarization and repolarization, results in a recordable, electric surface tension. It is assumed to be proportional to the effort of the measured muscle [11]. This particular recorder had a sampling frequency of 4000Hz and an amplifier bandwidth frequency of 5-1000Hz. The EMG signal above 400Hz (high pass filter) and below 10Hz (low pass filter) was filtered off and the frequencies in between were smoothed by the software (“proEMG”) of the recorder to the root mean square (RMS) as the unit being used in most EMG studies [3, 16]. As its name says, the RMS is the square root of the mean of the squared single signals. The electrode “Ambu Blue Sensor N” (20×30mm) provided a sensitivity of 600Ω -AC-impedance at an internal random noise of <15 μV.

The single leg hop has high validity and test-retest reliability in the quantification of strength and performance of the leg [18, 19]. Arm position was not prescribed [18]. Hop distance was always measured by the same person (M.K.) using the same measuring tape from the top of the hallux to the most posterior point of the heel. After one training hop, the second hop was measured and taken for further analysis.

### Analysis

EMG data were quantified by root mean squares (RMS) in microvolt (μV), single hop distance in centimeters (cm), and were described by their arithmetic means and standard deviations. Since intra-individual RMS differences were calculated, any possible systematic bias (e.g. by variable muscle activation patterns and quality of the skin between subjects) has been eliminated. To illustrate the activity of the agonist compared to that of the antagonist, the RMS ratio of the quadriceps (m. rectus femoris) to the antagonist (m. biceps femoris) was determined.

Intra-individual differences between two examinations were described and expressed as standardized response means (SRM). The SRM equals the group mean of the differences (between the tests with and without dorsal foot extension) derived from RMS-values or single leg hop distances for each individual divided by the standard deviation of those intra-individual differences of the group. Derived from the original Glass’s delta, the SRM is part of the family of effect sizes and quantifies the change of the whole group of participants (with minus without dorsal foot extension for each individual) in number of standard deviations of those changes [20–22]. It can be related to standardized mean differences of randomized controlled studies and is, in this sense, a modern standard of parametrizing effect sizes [22]. A positive SRM means improvement between two tests (with dorsal foot extension versus without dorsal foot extension). An SRM of 0.50-0.79 reflects moderate effects, an SRM ≥ 0.80 large effects.

To statistically detect at least a moderate effect, i.e. ≥half of the standard deviation of the differences, a minimum sample size of n ≥ 32 for intra-individual change data with an a priori type I error of *p* = 0.05 (5 %) and a power of 0.80 (type II error of 0.20) was required [21]. Since the changes of EMG and hop distances were symmetrically but only approximately normally distributed, the score changes were statistically compared by application of the Wilcoxon rank sum test. The non-parametric Wilcoxon test tests more conservatively than the *t*-test, i.e. significant results by the Wilcoxon test will also be significant by the *t*-test, but not vice versa. All analyses were performed using the statistical software SPSS 22.0 for Windows (SPSS Inc., Chicago, IL, USA).

## Results

### Subjects

The first 35 subjects who replied to the invitation and who met the inclusion criteria were recruited, whereby 3 of these were included in addition to the required 32 as a reserve. Twenty-three (66 %) of the 35 subjects were women. Mean age was 34.5 years (range 21-57years). Mean height was 170.7 cm (158-188 cm) and mean body weight was 65.2 kg (47-88 kg). They pressed a mean 12RM weight of 79.7 kg (40-160 kg) on the leg press.

### EMG activity without and with dorsal foot extension

The RMS (μV) without dorsal foot extension was relatively low and comparable for all 4 muscles at 45° knee flexion (Table 1). As indicated by the RMS increase for the tibialis anterior from mean 7.9 μV to 170.8 μV (SRM = 2.02), the activity of the m. vastus medialis with foot dorsal extension significantly grew by SRM = 1.39 and that of the m. rectus femoris by SRM = 0.71 (all p < 0.001).

**Table 1:** EMG activity data in RMS (μV) (*n* = 35)

RMS (μV)	– TA		+ TA		Difference		SRM	p
45° knee flexion	m	s	m	s	m	s		
m. biceps femoris	14.2	9.4	12.4	6.4	−1.8	8.9	−0.20	0.922
m. tibialis anterior	7.9	3.0	170.8	80.3	163.0	80.6	2.02	<0.001
m. vastus medialis	32.4	24.3	53.7	32.4	21.3	15.3	1.39	<0.001
m. rectus femoris	9.4	5.1	18.9	15.3	9.5	13.4	0.71	<0.001
90° knee flexion								
m. biceps femoris	23.0	13.4	28.9	16.3	5.9	6.1	0.97	<0.001
m. tibialis anterior	26.5	30.1	231.9	92.2	205.4	94.3	2.18	<0.001
m. vastus medialis	124.9	71.6	152.8	78.7	37.1	34.3	1.08	<0.001
m. rectus femoris	77.8	56.4	135.3	107.7	59.0	66.0	0.89	<0.001

Legend: RMS = root mean square of the electromyogram (EMG) in μV, *m* = arithmetic mean, *s* = standard deviation, SRM = standardized response mean (m of the difference(s) of the difference), *p* = type I error of the Wilcoxon test comparing the RMS with (+TA) to without dorsal foot extension (−TA)

At 90° knee flexion, all RMS levels were higher for all measurements (Table 1). Foot dorsal extension – proven by SRM = 2.18 for the m. tibialis anterior, which was comparable to that at 45° – resulted in a significant increase of SRM = 1.08 for the m. vastus medialis and of SRM = 0.89 for the m. rectus femoris (all p < 0.001).

The activity of the m. biceps did not increase at 45° (SRM = −0.20) but at 90° (SRM = 0.97). The mean RMS ratio of the rectus femoris to the biceps femoris increased from 0.66 without to 1.52 with foot dorsal extension at 45° knee flexion (computed from the values of Table 1). At 90° knee flexion, the ratio increased from 3.38 to 4.68.

### Single leg hop distance without and with dorsal foot extension

After dorsal foot extension on the leg press (see Fig. 1), mean single leg hop distance increased from a mean of 169.8 cm (at hop 2, before dorsal foot extension) to 178.9 cm (at hop 3, after dorsal foot extension) yielding an increase of 9.1 cm, SRM = 1.09 (p < 0.001) (Table 2). Between hop 1 and hop 2, leg press without dorsal foot extension was performed, which did not significantly alter the average hop distance (168.2 cm to 169.8 cm).

**Table 2:** Single leg hop distance (cm) data (*n* = 35)

	Distance		Difference			
	m	s	m	s	SRM	p
Hop 1 (−TA)	168.2	33.4				
Hop 2 (−TA)	169.8	32.9	1.6	10.5	0.15	0.518
Hop 3 (+TA)	178.9	35.1	9.1	8.4	1.09	<0.001

Legend: *m* = arithmetic mean, *s* = standard deviation, SRM = standardized response mean (m of the difference(s) of the difference), *p* = type I error of the Wilcoxon test

–TA = without activation of m. tibialis anterior, +TA = with activation of m. tibialis anterior. Between hop 1 and hop 2, EMG (−TA) was performed; difference in the line of hop 2 = difference hop 2-hop1, difference in the line of hop 3 = difference hop 3-hop2

## Discussion

This pilot study of healthy volunteers examined whether and to what extent EMG activity and hop performance of the m. quadriceps femoris changed without and with dorsal extension of the foot. Under foot dorsal extension, EMG activity of the m. vastus medialis increased by SRM = 1.39 (at 45° knee flexion on the leg press) and SRM = 1.08 (at 90°). The corresponding data for the m. rectus femoris were SRM = 0.71 and SRM = 0.89. All these changes were highly statistically significant and 3 of 4 reflected large effects (SRM ≥ 0.80). Consistently, the single leg hop distance highly significantly increased by SRM = 1.09 after foot dorsal extension, while it remained approximately constant for the two hops before dorsal foot extension.

This means that dorsal extension of the foot increased activity of the quadriceps musculature, which was consistently reflected by the objective EMG measurements at two knee flexion angles and the subjective, functional test. This effect seems to be at least partly specific to the m. quadriceps femoris since the EMG activity ratio rectus femoris to biceps femoris increased after foot dorsal extension at both knee flexion angles. The increase of EMG activity for the two tested quadriceps heads was also consistent. The m. vastus medialis is a single-joint head of the quadriceps and important for stabilization of the patella [23]. The m. rectus femoris is the two-jointed head of the quadriceps and plays an important role in the muscular balance of the ischiocrural muscle group, especially in knee stability affected by osteoarthritis [5, 14, 15, 24].

It could be proven that dorsal foot extension not only leads to increased electrophysiological activity but also to a progression of functional performance of the quadriceps musculature, a link that has previously only been hypothesized but not empirically tested [13, 14]. EMG activity remains a surrogate for functional performance. While some studies postulated a perfect correlation between muscle strength/performance and EMG activity, others evaluated rather low relationships [11, 25, 26].

As a theoretical construct, clinical experience and neuromuscular conditions lead to the hypothesis that knee extension is linked with simultaneous dorsal foot extension in most of the functional tasks as, for example, initiating a step forward. Both activities may form a neuro-functional chain of different muscles and the corresponding neural control. If this is so, simultaneous muscular activity may be linked in the corresponding motor-neural centers of the brain and the spinal cord. Specifically, dorsal foot extension can automatically increase tension and activity in the quadriceps, which increase effects of quadriceps training in training and rehabilitation. As a consequence, the quadriceps must work harder during dorsal foot extension at a constant weight load, e.g. the 12RM. This is supposed to result in higher training effects with lower weight loads and lower danger of injury and fatigue.

All relative EMG data differences (between with and without foot dorsal extension) were lower than those in the two comparative studies [13, 14]. Both studies had relatively low sample sizes: Tassi et al. *n* = 10, Teppermann et al. n = 20. Neither of them measured EMG of the m. tibialis anterior. The RMS levels of the vastus medialis were consistently higher in our study than those of the m. rectus femoris for all four measurements [13]. Finally, the finding that EMG activity of the m. rectus femoris decreases during extension of the knee (mean RMS from 90° to 45°: 77.8 μV to 9.4 μV without, 135.5 μV to 18.9 μV with foot dorsal extension) has been observed before [2, 14, 27]. The m. rectus femoris, together with its antagonist, the m. biceps femoris, which is more active the more the knee is flexed, stabilizes the knee during knee flexion [2, 27].

Strengths of the present study are the standardized setting of the test procedures and measurements according to recommendations of previous studies, the relatively (in comparison to other studies) high and a priori power-based sample size, and the combination of electrophysiological and functional measurement, which yielded consistent results.

Limitations are that the conclusions must be restricted to healthy, relatively young subjects. The sample size rated as absolutely low and prohibited stratified analyses. The results may have been influenced by fatigue of the subject during the testing process. However, measurements without dorsal foot extension were performed before those with dorsal foot extension. This aimed to reduce the difference between the two tests, i.e. bias by fatigue will tend to underestimate and not to overestimate the effects. For comprehensive measurement of all possible neuro-muscular chains, the m. gastrocnemius would have had to be examined by EMG as well. Absolute EMG is the measure used in most comparative studies, but RMS limits comparability between different subjects because of individual differences in muscle activation patterns and skin quality, for example. Future studies that aim to compare absolute EMG data across different subjects and populations should use normalized, relative EMG parameters as the maximal voluntary isometric contraction (MVIC).

## Conclusion

Activation of foot dorsal extension increased electro-myographic activation (as a surrogate for muscle strength) and functional performance of the m. quadriceps femoris. This finding can be used to improve effects in training and rehabilitation to increase functional muscle performance and knee joint stability. Future studies should prove whether short and long-term training effects under foot dorsal extension would be faster and higher for both healthy athletes and disease-affected knees such as those affected by osteoarthritis.

## Abbreviations

M: Musculus
EMG: Electromyography
RMS: Root mean square
RM: Repetition maximum
kg: Kilogram
cm: Centimeter
μV: Microvolt
Hz: Hertz
SRM: Standardized response mean

## References

[1] Tittel KM (2003). quadriceps femoris.

[2] Croce RV, Russell PJ, Decoster LC (2004). Knee muscular response strategies differ by developmental level but not gender during jump landing. Electromyogr Clin Neurophysiol.

[3] Escamilla RF, Fleisig GS, Zheng N, Lander JE, Barrentine SW, Andrews JR, Bergemann BW, Moormann CT (2001). Effect of technique variations on knee biomechanics during the squat and leg press. Med Sci Sports Exerc.

[4] Bizzini M, Biedert R, Maffiuletti N, Impellizzeri F (2008). Biomechanical issues in patellofemoral joint rehabilitation. Orthopade.

[5] Kelley Fitzgerald G, Piva SR, Irrgang JJ (2004). Reports of joint instability in knee osteoarthritis: Its prevalence and relationship to physical function. Arthritis Rheum.

[6] Da Silva EM, Brentano MA, Cadore EL, De Almeida AP, Kruel LF (2008). Analysis of muscle activation during different leg press exercises at submaximum effort levels. J Strength Cond Res.

[7] Reid KF, Pasha E, Doros G, Clark DJ, Patten C, Phillips EM, Frontera WR, Fielding RA (2014). Longitudinal decline of lower extremity muscle power in healthy and mobility-limited older adults: influence of muscle mass, strength, composition, neuromuscular activation and single fiber contractile properties. Eur J Appl Physiol.

[8] Hortobagyi T, Westerkamp L, Beam S, Moody J, Garry J, Holbert D, DeVita P (2005). Altered hamstring-quadriceps muscle balance in patients with knee osteoarthritis. Clin Biomech.

[9] Laube W, Hüter-Becker A, Dölken M (2005). Pathophysiologie, Muskelatrophie (pathophysiology, muscular atrophy). Biomechanik, Bewegungslehre, Leistungsphysiologie, Trainingslehre (biomechanics, kinematics, physiology of performance, training doctrine).

[10] Hart JM, Fritz JM, Kerrigan DC, Saliba EN, Gansneder BM, Ingersoll CD (2006). Quadriceps inhibition after repetitive lumbar extension exercise in persons with a history of low back pain. J Athl Train.

[11] Disselhorst-Klug C, Schmitz-Rode T, Rau G (2009). Surface electromyography and muscle force: Limits in sEMG-force relationship and new approaches for applications. Clin Biomech.

[12] Mau-Moeller A, Behrens M, Lindner T, Bader R, Bruhn S (2013). Age-related changes in neuromuscular function of quadriceps muscle in physically active adults. J Electromyogr Kinesiol.

[13] Tepperman SP, Mazliah J, Naumann S, Delmore T (1986). Effect of ankle position on isometric quadriceps strengthening. Am J Phys Med.

[14] Tassi N, Filho JG, Goncales M, Vitti M, Krool LB (1998). Electromyographic evaluation of the rectus femoris muscle during exercises performed on the leg press. Electromyogr Clin Neurophysiol.

[15] Nazario-de-Rezende F, Sousa G, Facury Neto MA, Bernardino R, Silva DC, Haddad EG, Goncales A, Santos LA (2006). Electromyographic study of the rectus femoris and biceps femoris (long head) muscles during bilateral isotonic contraction in a 45° leg press apparatus. Biosci J.

[16] Aarkskog R, Wisnes A, Wilhelmsen K, Skogen A, Bjordal JM (2012). Comparison of two resistance training protocols, 6RM versus 12RM, to increase the 1RM in healthy young adults. A single-blind, randomized controlled trial. Physiother Res Int.

[17] Baechle TR, Earle RW, Wathen D. Resistance training. In: Baechle TR, Earle RW (Eds.). Essentials of strength training and conditioning. National Strength and Conditioning Association. Human Kinetics 2000;18:395–425

[18] Ross MD, Langford B, Whelan PJ (2002). Test-retest reliability of 4 single-leg horizontal hop tests. J Strength Cond Res.

[19] Maulder P, Cronin J (2005). Horizontal and vertical jump assessment: reliability, symmetry, discriminative and predictive ability. Phys Ther Sport.

[20] Kazis LE, Anderson JJ, Meenan RF (1989). Effect sizes for interpreting changes in health status. Med Care.

[21] Angst F, Aeschlimann A, Stucki G (2001). Smallest detectable and minimal clinically important differences of rehabilitation intervention with their implications for required sample sizes using WOMAC and SF-36 quality of life measurement instruments in patients with osteoarthritis of the lower extremities. Arthritis Rheum.

[22] Borenstein M, Cooper H, Hedges LV, Valentine JC (2009). Effect sizes for continuous data. The Handbook of research synthesis and meta-analysis.

[23] Smith TO, Bowyer D, Dixon J, Stephenson R, Chester R, Donell ST (2009). Can vastus medialis oblique be preferentially activated? A systematic review of electromygraphic studies. Physiother Theory Pract.

[24] Coombs R, Garbutt G (2002). Developments in the use of the hamstring/quadriceps ratio for the assessment of muscle balance. J Sports Sci Med.

[25] Greenberger HB, Paterno MV (1995). Relationship of knee extensor strength and hopping test performance in the assessment of lower extremity function. J Orthop Sports Phys Ther.

[26] English R, Brannock M, Chik WT, Eastwood LS, Uhl T (2006). The relationship between lower extremity isokinetic work and single. J Sport Rehabil.

[27] Azegami M, Yanagihashi R, Miyoshi K, Akahane K, Ohira M, Sadoyama T (2007). Effects of multi-joint angle changes on EMG activity and force of lower extremity muscles during maximum isometric leg press exercises. J Phys Ther Sci.

